# Water Use Efficiency in Popcorn (*Zea mays* L. var. everta): Which Physiological Traits Would Be Useful for Breeding?

**DOI:** 10.3390/plants10071450

**Published:** 2021-07-15

**Authors:** Jhean Torres Leite, Antonio Teixeira do Amaral Junior, Samuel Henrique Kamphorst, Valter Jário de Lima, Divino Rosa dos Santos Junior, Kátia Fabiane Mereiros Schmitt, Yure Pequeno de Souza, Talles de Oliveira Santos, Rosimeire Barboza Bispo, Gabrielle Sousa Mafra, Eliemar Campostrini, Weverton Pereira Rodrigues

**Affiliations:** 1 Center of Agricultural Science and Technology, Laboratory of Plant Breeding, Darcy Ribeiro State University of Northern Rio de Janeiro, Av. Alberto Lamego, 2000, Campos dos Goytacazes 28013-602, RJ, Brazil; torresjhean@gmail.com (J.T.L.); valter_jario@hotmail.com (V.J.d.L.); juniorifagro@gmail.com (D.R.d.S.J.); kmedeirosschmitt@gmail.com (K.F.M.S.); yure_p-souza@hotmail.com (Y.P.d.S.); tallesdeoliveira@live.com (T.d.O.S.); rosimeirebarboza1@hotmail.com (R.B.B.); gabrielle.smafra@yahoo.com.br (G.S.M.); campostenator@gmail.com (E.C.); 2 Centro de Ciências Agrárias, Naturais e Letras, Universidade Estadual da Região Tocantina do Maranhão, Av. Brejo do Pinto, S/N, Estreito 65975-000, MA, Brazil; wevertonuenf@hotmail.com

**Keywords:** stress, photosynthesis, root angle

## Abstract

To ensure genetic gains in popcorn breeding programs carried out under drought conditions knowledge about the response of morphophysiological traits of plants to water stress for the selection of key traits is required. Therefore, the objective was to evaluate popcorn inbred lines with agronomically efficient (P2 and P3) and inefficient (L61 and L63) water use and two hybrids (P2xL61 and P3xL63) derived from these contrasting parents, cultivated under two water regimes (WW watered—WW; and water-stressed—WS) in a greenhouse, replicated five times, where each experimental unit consisted of one plant in a PVC tube. Irrigation was applied until stage V6 and suspended thereafter. Individual and combined analyses of variance were performed and the genotypic correlations and relative heteroses estimated. The water use efficient inbred lines were superior in root length (RL), root dry weight (RDW), and net CO_2_ assimilation rate (A), which were the characteristics that differentiated the studied genotypes most clearly. High heterosis estimates were observed for RL, SDW, leaf width (LW), leaf midrib length (LL), and agronomic water use efficiency (AWUE). The existence of a synergistic association between root angle and length for the characteristics A, stomatal conductance (gs), and chlorophyll concentration (SPAD index) proved most important for the identification and phenotyping of superior genotypes. Based on the study of these characteristics, the higher AWUE of the previously selected inbred lines could be explained. The results reinforced the importance of root physiological and morphological traits to explain AWUE and the possibility of advances by exploiting heterosis, given the morphophysiological superiority of hybrids in relation to parents.

## 1. Introduction

Climate change is associated with an increase in the concentration of greenhouse gases, disturbed rainfall patterns, increased air temperature, and can create an alarming scenario in terms of agricultural sustainability and food security [1,2,3,4,5]. In this context, drought was mentioned as the most harmful climatic incident for agricultural production, causing grain yield losses in maize fields of 20 to 30% in response to water stress during flowering and grain filling [6,7]. At the global level, limited water availability has become a serious problem in numerous tropical and subtropical climate regions [8,9,10]. In this context, water scarcity and increased temperature are highlighted as the main limiting factors of the full expression of the yield potential of agricultural crops [8,11,12].

Plants under water deficit in the soil are subjected to a series of morphological, physiological, biochemical, and molecular changes [13]. Some of these are decreased cell growth (reduction in leaf size and area), increase in abscisic acid content in roots, reduction in stomatal conductance, CO_2_ assimilation, and decreases in the enzyme activity related to nitrogen absorption, e.g., of nitrate and nitrite reductase. These physiological changes can greatly compromise agricultural productivity [14,15,16,17]. 

In regions with low rainfall, the characterization of genotypes is essential to support selection for more productive inbred lines, be they water stress tolerant, or water use efficient, thus mitigating the drought effects. Under soil water stress, the understanding of key characteristics for selection and of plant physiological processes is decisive to achieve genetic progress in plant breeding [18].

Aside from the frequently used characteristics to analyze plants with restricted water supply, as for example gas exchange and biomass production, the ability of these plants to develop a deeper root system in adaptation to soil water stress represents an agronomic and adaptive advantage [19]. In this sense, selection for a deep and extensive root system has been suggested to increase maize yields under water stress [20,21,22]. According to [23], the desirable root ideotype can be described as “steep, cheap, and deep”, indicating a greater angle of root growth in relation to the soil surface, lower metabolic expenditure for the development of the root system, and more efficient exploration of the soil subsurface. For maize inbred lines, [22] reported yield gains due to an intensified development of the root system, i.e., increases in dry weight and vertical extension, and to an improved water use efficiency. Therefore, studies that measure these traits associated with roots are fundamental for the selection of popcorn genotypes in environments with soil water deficit. 

In view of the above, the objective was to characterize the root phenotype and association with some physiological characteristics of popcorn inbred lines, previously selected as water use efficient and inefficient [24] and of their respective hybrids. Understanding the morphophysiological mechanisms associated with heterosis can create opportunities to increase the potential biomass production and, consequently, efficiently adapt plants to water stress.

## 2. Results

### 2.1. Effect of Water Restriction, Comparison between Genotypic Groups and Heterosis Estimates

#### 2.1.1. Morphological Characteristics

In the combined analysis, statistical differences were observed between genotypes (Gen) and water regimes (WR) for all morphological characters evaluated. There was no significant effect of the interaction genotype × water regime (Gen*WR) at *p* < 0.05 on the traits shoot dry weight/root dry weight ratio (ShDW/RDW), leaf weight (LW) and stem diameter (SD) (Table 1).

In the water use efficient inbred lines (WUEL) under water stress, the traits plant height (PH), leaf area (LA), stem dry weight (SDW), and lef dry weigh (LDW) were decreased by 46, 37, 22, and 54%, respectively. For the inefficient inbred lines (WUIL), the decrease for the same traits was 41, 24, 18, and 53%, respectively. For these traits, the decreases of these lines (together) were lower than of the hybrids (HY), for which the reductions were 42, 45, 32, and 59%, respectively, in the above order of traits. The shoot dry weight/root dry weight ratio (ShDW/RDW) was reduced by 4% in WUEL and 13% in WUIL. On the other hand, the ShDW/RDW ratio of the hybrids increased 18%. In addition, in WUEL water stress induced a reduction of 82, 9 and 17% in leaf weigh (LW), length of the leaf midrib (LL) and stem diameter (SD), respectively. In the WUILs, LW, LL, and SD, decreased by 59, 7, and 13%, respectively. For the same sequence of traits, decreases of 77, 9, and 16% were observed in the hybrids.

Under WS for the contrast WUEL × WUIL (C^1^), all characteristics except LA and LDW differed significantly (Table 1). In the contrast of means of hybrids versus inbred lines (C^2^), only PH and SDW had no significant differences at *p* < 0.05. For the other studied traits, the hybrid means were higher than those of the inbred lines. Relative heterosis (HET) of more than 10% was observed for SDW, LW, and LL (Table 1). The HET values of PH, LA, ShDW/RDW, and SD were low, with estimates below 9%.

Under WW conditions, the contrast of C^1^ means (WUEL versus WUIL) differed significantly for all characteristics except LA and LDW. For all morphological characteristics aside from ShDW/RDW and LW, WUEL had higher means. In the C2 mean contrast (inbred lines versus hybrids), only for PH and LW no significant differences were detected, demonstrating that the hybrids performed better for the other traits (Table 1). Relative heterosis above 10% was estimated for LA, SDW, ShDW/RDW, LW, LL, and SD.

#### 2.1.2. Physiological Characteristics

In the combined analysis, statistical differences were observed between genotype (Gen) and water regime (WR) for most of the physiological characters under study (Table 2). Exceptions were observed in Fv/Fm for source of variation Gen and agronomic water use efficiency (AWUE) for WR. There was a highly significant genotype × water regime (Gen*WR) interaction for the traits gs and Fv/Fm and significant for AWUE and WF (Table 2).

For efficient inbred lines (WUEL), water stress caused reductions of 65, 86, and 79% in net photosynthetic rate (A), stomatal conductance (gs), and transpiration rate (E), respectively. For WUIL, the reductions were 76, 87, and 72%, respectively, for the same characteristics. The hybrids were affected by reductions of 86, 88, and 87% for A, gs, and E, respectively. In the mean, the chlorophyll concentration (SPAD index), maximum quantum efficiency of photosystem II (Fv/Fm) and water footprint (WF) of WUELs decreased by 13, 3, and 8%, respectively. For WUILs, the reductions in SPAD index, Fv/Fm and WF were 11, 11, and 14%, respectively. For the hybrids, the reductions of these same characteristics were 11, 4, and 4%, in the same order. For leaf-to-air vapor pressure difference (VPDleaf-air) and leaf temperature (TL), mean increases of 30 and 2% were observed, respectively, for WUEL. For WUIL, the increases were 23 and 2% for VPDleaf-air and TL, respectively. For the hybrids, the increases for the same traits were 33 and 4%, respectively.

Under WS conditions, the C^1^ mean contrast analysis (WUEL × WUIL) indicated significant differences for all characteristics, except for E and WF (Table 2). Characteristics A and gs were superior in WUEL. In the contrast of C^2^ means, the characteristics TL, Fv/Fm and SPAD did not differ significantly at *p* < 0.05. For the hybrids, VPDleaf-air was significantly higher. Analyzing the relative heterosis under this water regime, high and negative estimates were observed for A, gs and E, respectively, of −47%, −31%, and −49% (Table 2). In addition, heterosis of −11% was observed for water footprint (WF) and high positive HET values (12%) for AWUE.

Under WW conditions, the C^1^ mean contrast indicated significant differences for only the variables AWUE and WF (Table 2). In the contrast of C^2^ means, a significant difference was observed only for gs, AWUE, and WF. High relative heterosis, greater than 20%, was observed for gs and WF, in the negative direction, and in AWUE, in the positive direction (Table 2).

#### 2.1.3. Root Traits

For the water use efficient inbred lines (WUEL), water restriction led to reductions of 11 and 31% for root length (RL) and root dry weight (RDW); and in water use inefficient inbred lines (WUILs) to 8 and 40%, respectively, for the same characteristics. For the hybrids, RL and RDW decreased by 13 and 49%, respectively. For root architecture, support root angle (SRA) and crown root angle (CRA) increased by 25 and 34% in WUEL, while for WUIL, the reductions were 12% for the same two characteristics. In the hybrids, SRA and CRA were reduced by 0.30 and 18%, respectively. The traits number of support roots (NSR) and number of crown roots (NCR) decreased at the same intensity in all genotype groups. Averaged across the genotype classes, mean reductions of 26 and 3% were observed for support root density (SRD) and crown root density (CRD).

Under WS, in the contrast of C^1^ means (WUEL versus WUIL), only RL and RDW differed significantly (Table 3). For most characteristics, the WUEL means tended to be higher than those of the WUIL, except for CRA and NCR (Table 3). In the contrast of C^2^ means, only CRA differed significantly. Hybrids tended to have higher means for RDW, SRD, and NCR than the inbred lines. The highest values of relative heteroses, in general, tended to be negative, as observed for SRA, NSR and CRA (−13, −11, and −23%, respectively). For SRD, positive heterosis was high (14%).

Under WW conditions, for the C^1^ mean contrast, RL, SRA, CRA, and CRD differed significantly (Table 3). In the contrast of C^2^ means, a significant difference was only detected between RDW and CRA. The characteristics RL and RDW had high relative heterosis (around 10 and 20%, respectively). The values of the other characteristics were rather low, with heterosis estimates below 10%.

### 2.2. Genotypic Correlations

**Figure 1:**
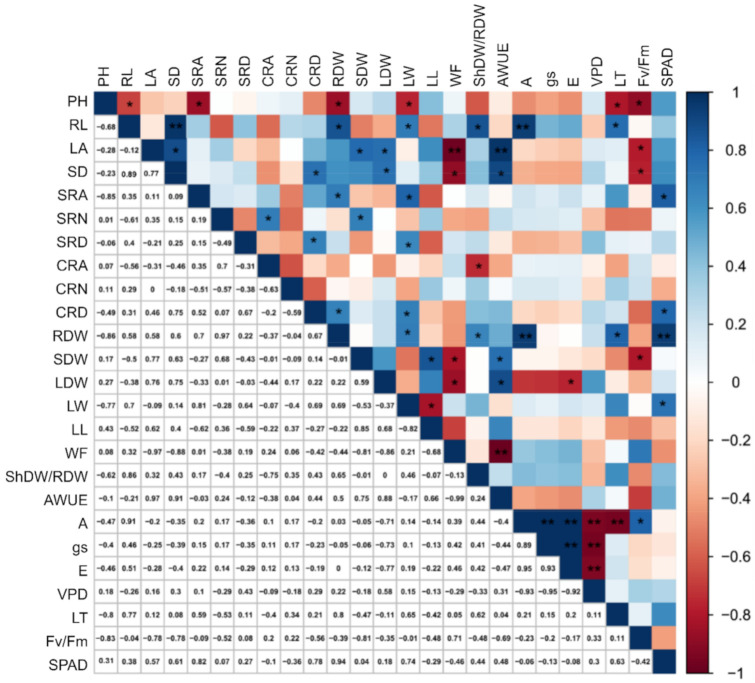
Estimates of genotypic correlations between shoot, physiological and root morphological characteristics in popcorn under water stress. PH = plant height; LA = leaf area; SDW = stem dry weight; LDW = leaf dry weight; ShDW/RDW = shoot dry weight/root dry weight ratio; LW = leaf width; LL = length of the leaf midrib; SD = stem diameter; A = net photosynthetic rate; gs = stomatal conductance; E = transpiration rate; VPD leaf/air = vapor pressure deficit; TL = leaf temperature; Fv/Fm = chlorophyll fluorescence; SPAD index = relative chlorophyll content; AWUE = agronomic water use efficiency; WF = Water footprint; RL = root length; RDW = root dry weight; SRA = support root angle; NSR = number of support roots; SRD = support root density; CRA = crown root angle; NCR = number of crown roots; and CRD = crown root density. * = significant difference at 5%; and ** = significant difference at 1% by the Pearson correlation test.

Analyzing the genotypic correlation coefficients (rg) under water-stressed (WS) (Figure 1), for plant height (PH), significant and negative values were estimated for the characteristic root length (RL) (−0.68), support root angle (SRA) (−0.85), root dry weight (RDW) (−0.86), leaf weight (LW) (−0.77), leaf temperature (TL) (−0.80), and Fv/Fm (−0.83). Characteristic RL expressed a significantly positive estimate of rg for length of the leaf midrib (LL) (0.79), stem dry (SD) (0.89), RDW (0.58), LW (0.70), ShDW/RDW (0.86), net photosynthetic rate (A) (0.76) and TL (0.78). Trait leaf area (LA) had a positive and significant rg for SD (0.89), stem dry weight (SDW) (0.78), leaf dry weight (LDW) (0.76), and agronomic water use efficiency (AWUE) (0.97); and negative for PH (−0.97) and Fv/Fm (−0.78). 

The variable SD correlated positively with CRD (0.75), LDW (0.75) and AWUE (0.90); and negatively for PH (−0.90) and Fv/Fm (−0.78). The trait SRA had a positive and significant correlation with RDW (0.70), DF (0.80) and SPAD index (0.82). The characteristic NSR was positively correlated with CRA (0.70) and RDW (0.68) and SRD significantly and positively correlated with CRD (0.67) and LW (0.64). Variable CRA was negatively correlated with ShDW/RDW (−0.75) and CRD significantly and positively with RDW (0.67) and LW (0.69). For RDW, there was a significant and positive correlation in the association with LW (0.57), ShDW/RDW (0.66), A (0.97), TL (0.80), and SPAD (0.94). Trait SDW was positively correlated with LL (0.85) and AWUE (0.75) and negatively with WF (−0.81) and Fv/Fm (−0.81). LDW positively correlated with the AWUE (0.89); and negatively for WF (−0.86) and E (−0.77). Leaf width correlated positively with SPAD (0.74) and negatively with LL (−0.83). The ratio WF correlated positively with AWUE (−0.99). The trait net photosynthetic rate (A) was positively correlated with gs (0.95) and E (0.93), in addition to the previously mentioned RL and RDW. On the other hand, the estimates of A were a negative for TL (−0.95) and VPD (−0.92). The correlation of gs with E (0.99) was positive and with VPD negative (−0.95), both with high values (Figure 1).

**Figure 2:**
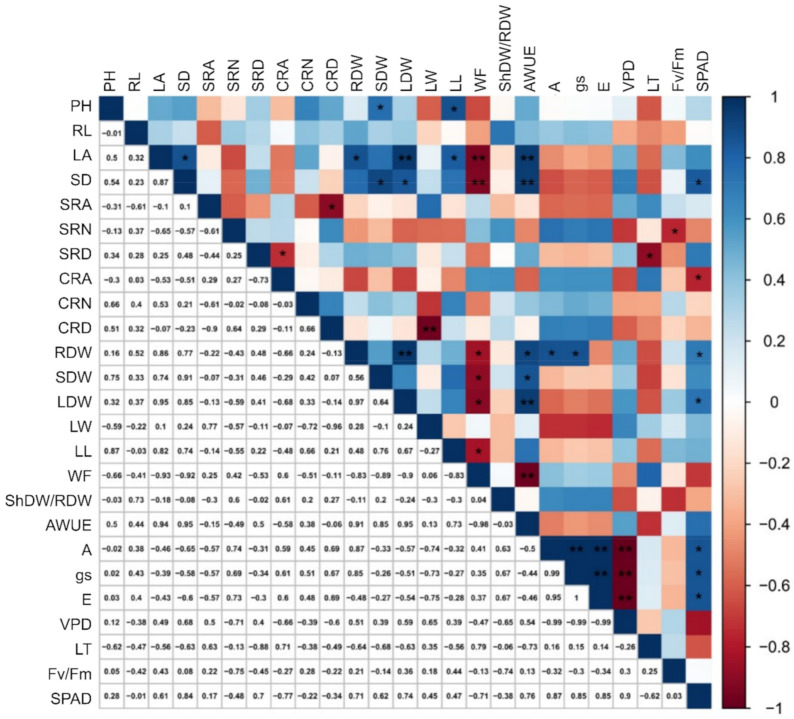
Estimates of genotypic correlations between shoot, physiological and root morphological characteristics in popcorn under well-watered conditions. PH = plant height; LA = leaf area; SDW = stem dry weight; LDW = leaf dry weight; ShDW/RDW = shoot/root ratio; LW = leaf width; LL = length of the leaf midrib; SD = stem diameter; A = net photosynthetic rate; gs = stomatal conductance; E = transpiration rate; VPDleaf/air = vapor pressure deficit; TL = leaf temperature; Fv/Fm = chlorophyll fluorescence; SPAD index = relative chlorophyll content; AWUE = agronomic water use efficiency; B/W = ratio of biomass production to water consumption; RL = root length; RDW = root dry weight; SRA support root angle; NSR = number of support roots; SRD = support root density; CRA = crown root angle; NCR = number of crown roots; and CRD = crown root density. * = significant difference at 5%; and ** = significant difference at 1% by the Pearson correlation test.

In the WW cultivation condition, PH had a significant and positive correlation with SDW (0.76) and LW (0.87) (Figure 2). Trait LA was significantly and positively correlated with SD (0.87), RDW (0.86), LDW (0.95), LW (0.82), and AWUE (0.94); and negatively with WF (−0.93). The characteristic SD showed positive correlations with SDW (0.90), LDW (0.85), AWUE (0.95) and SPAD (0.84) and a negative correlation with PH (−0.93). The trait support root angle (SRA) had a significant and negative correlation only with CRD. For number of support roots (NSR), a negative correlation was observed for Fv/Fm (−0.75). Support root density expressed negative correlations with CRA (−0.73) and TL (−0.88). The CRA characteristic was negatively correlated with SPAD (−0.77). For CRD, the correlation was only negative for LW (−0.96). For RDW, correlations were positive with LDW (0.97), AWUE (0.91), A (0.87), gs (0.85), and SPAD (0.71), and negative with PH (−0.84). Stem dry weight (SDW) correlated positively with AWUE (0.85) and SPAD (0.74); and negatively with WF (−0.89). Leaf dry weight correlated positively with AWUE (0.95) and negatively with WF (−0.90). Leaf length had a negative correlation (−0.83) only with WF. WF was shown to be negatively correlated (−0.98) with AWUE. For net photosynthetic rate (A), significantly positive correlations with gs (0.99), E (0.99) and SPAD (0.87) were observed; and negative correlations only with VPD (−0.99). The variable gs correlated positively with E (0.99) and SPAD (0.85) and negatively with VPD (−0.99). The transpiration rate (E) correlated positively with SPAD (0.85) and negatively with VPD (−0.99) (Figure 2). 

## 3. Discussion

The application of water stress negatively affected most characteristics, the morphological traits associated with shoot and root as well as the physiological characteristics, and (as expected) resulted in higher means under well-watered (WW) than under water-stressed (WS) conditions. This was due to the greater differentiation of the genotypes under WS, which could also lead to higher heritability values. In fact, [25] showed that popcorn genotypes under drought in the field had higher heritability, which indicates greater reliability of selection in this water regime, due to greater expression of genetic variance. On the other hand, in general, the WS regime allowed better genotypic discrimination. In relation to the studied genotypic groups in both water regimes (WR), the inefficient inbred lines (WUIL) had lower means for the characteristics plant height (PH), leaf area (LA), leaf dry weight (LDW), length of the leaf midrib (LL) and stem diameter (SD) and higher mean estimates for shoot dry weight/root dry weight ratio (ShDW/RDW), leaf weight (LW) and water footprint (WF). Under both WR, higher values of LA of efficient inbred lines (WUEL) were associated with a larger surface area to intercept solar radiation and, consequently, increase photosynthetic carbon assimilation (A), since higher stomatal conductance (gs) was observed in WUEL plants. Higher gs values improve the transfer of CO2 to Rubisco carboxylation sites and can therefore increase A and biomass production [26]. Reinforcing the previous arguments, a high correlation of A to root dry weight (RDW) was observed in this study, in both water regimes.

Under both WR, leaf area (LA) was the most important characteristic for the differentiation between inbred lines and hybrids, since the hybrids had significantly higher LA than the inbred lines. Moreover, for both WR, dry biomass of stem, leaf, and root (TDW) were highest in WUEL. The hybrids of the WUEL plants achieved the highest means, indicating an opportunity to explore heterosis based on these characteristics. According to [27] the accumulation of dry matter before flowering and the consequent increase in surface area for interception of solar radiation (>leaf area) can explain the agronomic advantages in terms of production under drought conditions. Furthermore, in the sense of exploiting heterosis, [28] showed that the metabolic superiority of the hybrids over the inbred parents may be due to the larger leaf area of the hybrids. In addition, a higher shoot biomass is associated with deeper root systems and a greater angle of the support root in relation to the soil surface [29], which may allow improved soil exploration under drought. In fact, in this study, higher root biomass was associated with higher aboveground biomass, regardless of the WR (Figure 1 and Figure 2).

In the comparison of WRs, the WUIL had lower proportional losses, while on the other hand, they produced less shoot biomass (PH, LA, LDW, LL, and SD). According to [30], a greater shoot biomass requires greater amounts of water. This explains the higher water consumption by WUEL and hybrid parents, which had higher shoot and root biomass. In this sense, it is worth emphasizing that the inbred lines were pre-selected for agronomic water use efficiency (g L-1), in other words, the inbred lines that best converted the absorbed water into grain yield, which allowed a more accurate genotypic discrimination, in the field as well as in the greenhouse.

Under drought, WUIL had a higher ShDW/RDW ratio, i.e., a greater shoot than root biomass, which means that less photoassimilates were invested in the roots of these genotypes to explore larger extensions of soil. This variable has been mentioned as important for selection of drought-tolerant maize genotypes [31]. In this sense, the WUEL parents and hybrids stood out with lower estimates of the ShDW/RDW ratio, which shows the greater metabolic investment in the root than in the shoot parts. 

Regardless of the WR, for most of the morphological shoot variables (LA, LDW, ShDW/RDW, LL, and LW), the hybrids had higher means than the inbred lines. Several authors have suggested the influence of heterosis on better adaptation and higher production of plants in environments under abiotic stresses [32] or fully saturated conditions [33]. To reinforce these arguments, some authors emphasize the ability of hybrids to maintain cell homeostasis and the complete functioning of plant metabolism under abiotic stress, indicating an adaptive advantage to adverse situations compared to the parents [30]. Another explanation for the superiority of hybrids over their parents was shown by [34], who claims that the greater efficiency in the use of available energy resources is associated with a reduction in enzyme activity, in addition to a greater stability of structural proteins. In this sense, regardless of the greater reductions from WW to WS observed for hybrids, in a separate analysis of each WR, the morphological characteristics of the hybrids are superior.

The WUIL parents had greater proportional reductions between WR for the variables A, gs, VPDleaf-air, and Fv/Fm, compared to WUEL and the hybrid parents. The decrease in A and gs and the increase in VPDleaf-air were associated with a reduction in gas exchange due to the stomata closure in response to water restriction [35]. These characteristics are affected by root water acquisition and can significantly control biomass production [36]. As reported by [37], the correlations between physiological and morphological variables, above all, net photosynthetic rate with dry and fresh root and shoot weight were significant and high. On the other hand, in an evaluation of maize hybrids, [32] obtained a high R2 estimate (0.80) in the association between total biomass and grain yield. These reports corroborate the results of this work, in which the correlation between the root system (root growth angle and root length development) and biomass production proved important for the discrimination of more productive genotypes under field conditions. For the variable A, which is influenced by non-stomatal effects, the reduction in Fv/Fm estimates to below 0.75 may be related to a limitation of the photosynthetic process by excess light and damage caused to the D1 protein of photosystem II (PSII) [38]. In this study, all Fv/Fm estimates were above 0.75, which shows that even under severe water limitation, the photochemical machinery of popcorn plants was not compromised, which is why the reduction in A can be attributed to stomatal conductance effects. In this condition of soil water limitation, in which values above 0.75 were maintained, excess excitation energy may have been dissipated in other ways, due to an alternative electron flow [39,40].

The results of this research show drastically reduced gas exchange rates in WUIL parents under WS, which consequently compromised CO2 assimilation by stomatal effects. The reduction in leaf chlorophyll concentration (SPAD index) did not affect the photochemical efficiency of photosystem II (PSII), when it was evaluated by the Fv/Fm ratio [41]. This information was inferred from the negative correlations between Fv/Fm, TL, VPDleaf-air and A, as well as between Fv/Fm, VPDleaf-air and gs (Figure 1 and Figure 2). On the other hand, the inbred lines expressed higher means of gas exchange and VPDleaf-air in relation to the hybrids under WS. The reason may be the higher biomass of the hybrid shoots, which require greater water resources and may therefore have resulted in greater losses under dry conditions. However, although a greater decrease in photosynthetic efficiency was observed in the hybrids, the exact causes and consequences of the original predisposition to faster growth and biomass accumulation differed even among the hybrids. A better performance of the photosynthetic system of inbred lines compared to maize hybrids was reported by [30], who found higher values of stomatal conductance, net CO2 assimilation rate and transpiration rate of the inbred lines. Nevertheless, the morphological development of the hybrids was more intense than that of the inbred lines. 

Higher values of root length (RL) and root dry weight (RDW), as observed in the WUEL parents can ensure a greater potential soil exploration in the case of drought. In fact, the characteristics of root architecture (root length and number of crown roots) can increase the soil volume explored for water and nutrient uptake, which can increase production in environments under abiotic stresses, as described by [42]. Some studies found that root length, biomass and density can be considered characteristics of interest for the selection of genotypes under soil water stress, for allowing greater soil exploration in lateral and vertical directions [43,44,45]. It is no coincidence that these characteristics are related to greater water use efficiency in popcorn [46]. Among mature maize plants in the field, the most productive were the genotypes with the highest RL and RDW [29,47]. Therefore, the potential of exploration of deeper soil strata proved to be an important characteristic for the selection of genotypes for water-limited environments [22,42]. 

Regardless of the studied genotype group, an adaptation of the root angles under WS was observed, with increases in SRA and CRA. This means that steeper roots with straighter profiles are more adaptable to draw water from deeper soil layers. Genotypes with these root characteristics may have advantages in the exploration of subsurface soil layers, allowing greater water uptake [18,46,48]. Under drought conditions, [19] showed that the most productive genotypes were those with deeper roots. Root length was the trait that differentiated the WUEL from WUIL genotypes most clearly, regardless of the WR. Apparently, a longer RL improved the adaptation of the efficient inbred lines to water stress. In addition, in a comparison of plants under both WR, of both parent groups WUEL and WUIL, those with greater RL also had a greater increase in SRA and CRA. The synergistic association of these two characteristics resulted in significant correlations in positive direction between SDW and LW for SRA, and negative direction between PH and ShDW/RDW for CRA. In fact, [49] reported that in maize inbred lines and hybrids, those with highest biomass were the genotypes with the greatest root growth angle (SRA and CRA) in relation to the soil surface. Nevertheless, Lynch (2013) reported that a greater angle of crown and support roots in relation to the soil surface, associated with greater root length, was correlated with higher grain yield values. Heterosis effects were highest for traits related to shoot biomass as well as plant height and leaf and root area, especially under WS conditions. Accordingly, some studies explained the greater superiority of hybrids by the more marked expression of morphological characteristics related to shoot and root, as well as to traits associated with the photosynthetic machinery [32,33,37,50,51]. In this sense, the characteristics RL, RDW, and SRD stand out, for which high values of relative heterosis were observed. However, under water stress, this study showed that hybrids may be subjected to a higher level of damage regarding the photosynthetic process, regardless of the greater development of shoots and roots. 

Therefore, the use of beneficial effects of heterosis for breeding programs for water restriction is proposed, since the estimates of LA, SDW, LDW, RDW, LL, and SD, as well as ShDW/RDW ratio of the evaluated hybrids were higher. This led to the conclusion that heterosis in the characteristics related to the increase in shoot and root biomass contributed to the greater hybrid growth, mainly when under water stress. The effects of heterosis in morphological development were also observed under well-watered conditions, although at a lower intensity. In this sense, the response of hybrids, especially in the reproductive phase, tends to mitigate the loss in crop yield. 

## 4. Materials and Methods

Four S_7_ popcorn inbred lines previously selected as water use efficient (P2 and P3, from the compound CMS-42) and inefficient (L61 and L63, from the BRS-Angela population) [18,52], as well as two hybrids of the cross between these four inbred lines (P2xL61 and P3xL63) were used for evaluation under different water regimes. The experiment was carried out in a greenhouse, at a research base of the State University of Norte Fluminense Darcy Ribeiro, in Campos dos Goytacazes, Rio de Janeiro, Brazil (21°45′45″ S 41°19′6″ W). 

Each experimental unit consisted of one PVC tube (diameter 0.20, length 1.00 m), with one plant per tube. The bottom of the tubes was closed and they were filled with substrate consisting of a 1:1 soil/sand mixture. The top of the tube was not covered to allow water to vaporize through evaporation. The substrate was previously analyzed and limed according to the nutritional needs of the crop [53]. A NPK fertilizer mixture (04-14-08) was applied at a rate of 250 kg ha^−1^, that is, 25 g per tube. Three seeds were germinated and thinned to one plant per tube 15 days after sowing. 

The experimental water regimes (WR) were characterized as follows: (i) full irrigation (WW—Well-watered), at field capacity (−0.01 MPa); and (ii) water deficit (WS—water-stress condition—induced by suspension of irrigation at 30 days after sowing until reaching the water potential of −1.5 MPa, and maintained at this level or below the wilting point until the end of the experiment (45 days after sowing). The soil water potential was monitored with MPS-6 sensors (DECAGON^®^, Pullman, WA, USA) at a depth of 50 cm, in two samples of each WR. Moisture was adjusted by a drip irrigation system, with a dripper flow rate of 4 L h^−1^ and a timer to adjust the irrigation pattern and water amount. From the 5th day after sowing onwards, water was applied five times a day, i.e., a total of 420 mL of H_2_O tube^−1^ day^−1^.

The plant-available water was calculated based on the evapotranspiration rate and maize crop coefficient Kc (0.5) [54]. In addition to this information, for the calculation of water application estimates, the volume of the container (0.0314 m^3^) and area of the wet bulb formed by drip irrigation in the soil (30 cm deep) were also considered.

All plants were grown under full irrigation until the phenological stage V6 (six nodes on main stem), and thereafter, irrigation was suspended. Under WS, no irrigation was applied between stage V6 and V12 (45 days). During the entire period, treatment WS was watered with 10.80 L plant^−1^ and WW with 18.90 L plant^−1^.

**Figure 3:**
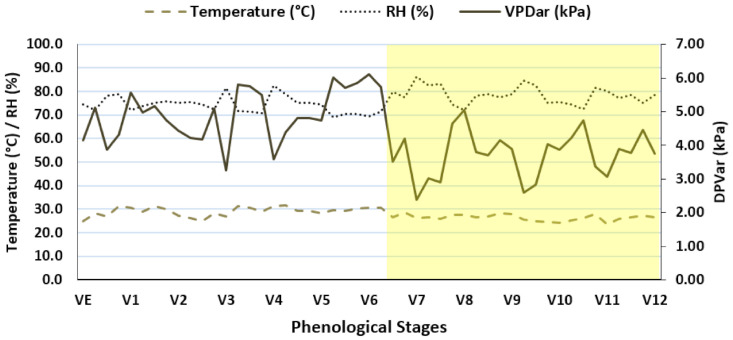
Means of temperature, relative air humidity, and vapor pressure deficit in the experimental period for each phenological stage of the crop. The yellow area indicates the period without irrigation (45 days).

The experiment was arranged in a randomized block design with six genotypes and two water regimes, with five replications. Weather conditions were monitored and recorded on a USB Data Logger Extech RHT10. Additionally, incident irradiance was measured with a MultispeQv1.6 light sensor (Photosynq inc., East Lansing, MI, USA). In general, the temperature ranged from 19.10 to 44.40 °C and the mean relative humidity was 76.48% (Figure 3). The solar irradiance sensor recorded 700 µmol.m^−2^ s^−1^ in the period of highest light intensity. The values of microclimatological variables were expressed as a result of the phenological stages of the crop (Figure 3).

**Figure 4:**
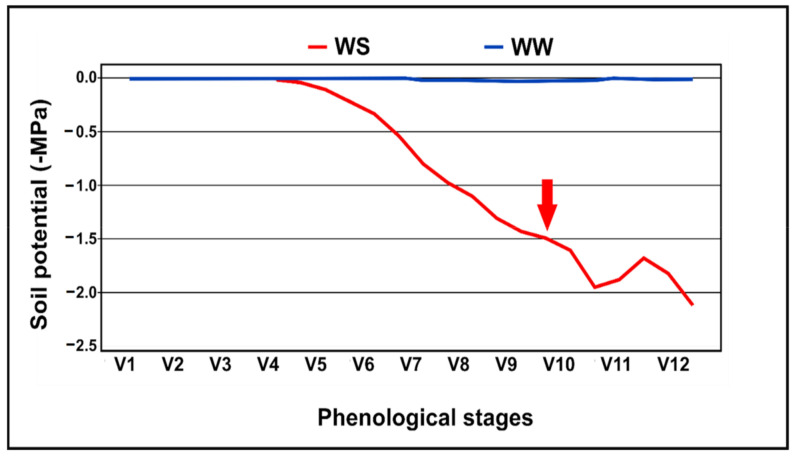
Soil water potential during the development of popcorn inbred lines and hybrids, according to the phenological stage, under two water regimes. WS—water stressed; WW—well-watered. The red arrow represents the time when the permanent wilting point was reached in the WS treatment, i.e., −1.5 MPa.

Under the WS regime, the soil water potential ranged from −0.1 to −1.6 MPa, from planting to the end of the experiment, and the plants reached −1.5 MPa at stage V9 (after 30 days) and were maintained at this level until harvest. On the other hand, in the WW regime, the soil water potential was maintained at field capacity (−0.1 MPa) (Figure 4).

### 4.1. Study Traits

#### 4.1.1. Morphological Traits

At harvest, the plant shoots were measured to determine plant height (PH), measured in cm from the soil surface to the ligule of the last developed leaf; the leaf area (LA), in m2, was estimated with a portable leaf area meter (Li-3100, LiCor, Lincoln, NE, USA); stem dry weight (SDW), lead dry weight (LDW), and root dry weight (RDW), for which the stalks and leaves were placed in paper envelopes and dried at 70 °C for 72 h. The shoot dry weight (ShDW) (ShDW = SDW + LDW) was used to calculate the ShDW/RDW ratio. Leaf width (LW) was measured with centimeter ruler, in the middle third of the third fully developed leaf. The length of the leaf midrib (LL) was measured from the base to the tip of the third fully developed leaf and the stem diameter (SD) at about 10 cm above the soil surface with a digital caliper (Digital Vernier Caliper (0–150mm), Brazil).

#### 4.1.2. Physiological Traits

All physiological variables were measured at harvest. The relative leaf chlorophyll concentration (SPAD index) was estimated using a portable chlorophyll meter (SPAD 502, Minolta Company, Osaka, Japan).

Gas exchange was assessed with an infrared gas analyzer—IRGA (LI-6400, Li-Cor, Inc., Lincoln, NE, USA) in a controlled atmosphere (leaf temperature of 25 °C, 400 μmol mol^−1^ CO_2_ and 700 μmol m^−2^ s^−1^ photosynthetic active radiation) in a 2 × 6 cm chamber. The variables net CO2 assimilation rate (A), transpiration rate (E), stomatal conductance (gs), and leaf-to-air vapor pressure difference (VPDleaf-air) were measured with the LI6400. To assess the maximum quantum efficiency of photosystem II (Fv/Fm), a portable fluorimeter model MultispeQv1.0 (Photosynq inc., USA) was used. Gas exchange and chlorophyll fluorescence emissions were measured in the middle third of the fourth (fully developed) leaf from the apex, between 08:00 and 11:00.

Leaf temperature (TL) was evaluated with a thermographic camera (model i50, FLIR, USA) and the images analyzed with FLIR QuickReport. The pictures were taken 60 cm from the plant apex, at an angle of 45° between the thermograph and plant apex, between 12:00 and 13:00.

The agronomic water use efficiency (AWUE, g L H_2_O^−1^) was calculated as the ratio of the total dry biomass production (ShDW+RDW) by the water volume applied to each plant throughout the experiment, and the water footprint (WF, in L H_2_O g^−1^) was computed as l/ShDW+RDW.

#### 4.1.3. Root Traits

At 45 days after planting, the culture tubes were emptied. Initially, the substrate was separated from the roots by slight shaking and then the roots were washed under tap water to remove the rest. Afterwards, the roots were rinsed with distilled water and the surface dried with a paper towel. Root length (RL) was measured from the surface of the root apex to the intersection with the plant stem, and root dry weight (RDW) after drying in a forced ventilation oven at 70 °C for 72 h. The angles and density of the support root (SRA and SRD) and of the crown root, respectively (CRA and CRD) were evaluated with a ruler for maize root phenotyping (Maize Shovelomics Scoreboard) [18]. The number of support roots (NSR) and number of crown roots (NCR) were also counted.

### 4.2. Statistical Analyses

Combined and individual analyses of variance were performed to decompose the variation between genotypes (Gen) and water regime (WR), as well as the effect of the interaction genotype × water regime (Gen*WR). The following statistical model was used for individual analysis individual: Y_ij_ = µ+ G_i_ + B_j_ + E_ij_, where: Y_ij_ is the value of the plot associated with the i-th genotype in the j-th replication; µ the general parametric mean of the data under study; G_i_ the i-th genotype effect; B_j_ the effect of the j-th replication and E_ij_ the experimental error associated with observation Y_ij_. A posteriori, the sources of genotype variation and water regime with fixed effects were taken into consideration and combined analysis was performed based on the statistical model: Y_ijk_ = µ+ G_i_ + [CH]j + B/[CH]_jk_ +[GCH]_ij_ + E_ijk_, where: Y_ijk_ is the phenotypic value measured in the i-th genotype in the j-th water regime in the k-th replication; µ = overall parametric mean of the data under study; G_i_ = effect of the i-th genotype; [WR]_j_ = effect of the j-th water regime; B/[WR]_jk_ = eblock effect within water regime; [GWR]_ij_ = interaction effect of the i-th genotype with j-th water regime; and E_ijk_ = mean error associated with observation Y_ijk_. The analyses were performed with the computer program GENES [55].

Consequently, the line effects were decomposed according to the notation WUEL × WUIL (water use efficient × water use inefficient inbred lines) and inbred lines * hybrids, for each trait. Subsequently, the genotypic correlations were estimated and tested at the level of 5 and 1% of probability, according to the *t* test. Software SAS ^®^ (Version 8, SAS Institute Inc., Cary, NC, USA, 2002) was used for the analyses.

### 4.3. Heterosis

The relative heterosis of each hybrid was estimated by the expression:

h__ij_ (%) = (¯(S__ij_) − ¯(P__ji_))/¯(P__ji_) × 100, (1)

where:

h__ij_ (%): heterosis percentage of the cross of the j-th inefficient with the efficient line ‘i’;

¯(S__ij_): mean of the hybrid derived from the inefficient line ‘j’ with the efficient line ‘i’; and

¯(P__ji_): mean of the parents of the j-th inefficient line with the efficient line ‘i’.

## 5. Conclusions

The morphophysiological variables discriminated the difference between the studied genotypes with greater precision under WS, which may indicate a greater capacity for the selection of water use efficient genotypes. The growth of the root system (RL and RDW), gas exchange and photochemical efficiency of photosystem II of the efficient inbred lines were greater. The synergistic association between root related characteristics and characteristics associated with the photosynthetic process showed the importance of including physiological assessments to detect genotypic superiority and revealed heterosis as a result of biochemical processes. 

## Figures and Tables

**Table 1:** Summary of analysis of variances, mean estimates, standard deviations and relative heteroses of the decomposition of effects between genotypic groups (WUEL, WUIL, and HY) in popcorn for morphological characteristics evaluated under different water regimes.

Trait	Combined Analysis			Water-Stressed						Well-Watered					
	Gen	WR	Gen * WR	WUEL	WUIL	C^1^	HY	C^2^	HET	WUEL	WUIL	C^1^	HY	C^2^	HET
PH	**	**	*	79.12 ± 4.20	68.8 ± 1.80	**	78.9 ± 5.36	ns	7.87	147.5 ± 18.54	116.2 ± 7.58	**	136.10 ± 14.00	ns	3.69
LA	**	**	**	0.22 ± 0.01	0.21 ± 0.01	ns	0.27 ± 0.01	**	4.89	0.36 ± 0.01	0.28 ± 0.01	ns	0.44 ± 0.02	**	18.10
SDW	**	**	**	7.00 ± 1.48	12.01 ± 2.16	*	13.80 ± 0.69	ns	20.8	31.32 ± 2.42	25.53 ± 5.48	**	33.32 ± 5.19	***	42.60
LDW	**	**	*	13.35 ± 1.29	12.41 ± 1.94	ns	15.56 ± 1.93	***	−5.05	17.05 ± 2.21	15.14 ± 2.85	ns	22.93 ± 2.89	***	7.09
ShDW/RDW	**	**	ns	4.03 ± 0.03	4.15 ± 0.03	**	4.50 ± 0.03	**	7.31	3.66 ± 0.08	4.6 ± 0.06	*	4.43 ± 0.06	*	18.01
LW	**	**	ns	16.67 ± 1.25	17.99 ± 1.62	**	4.60 ± 9.06	**	13.4	36.59 ± 6.53	44.49 ± 8.30	**	43.41 ± 6.87	ns	14.50
LL	**	**	*	92.55 ± 6.27	70.1 ± 2.76	***	92.01 ± 4.86	***	10.6	101.3 ± 7.37	75.3 ± 5.99	***	101.10 ± 5.71	***	37.10
SD	**	**	ns	14.56 ± 0.06	14.4 ± 0.07	**	15.77 ± 0.93	**	8.83	17.51 ± 0.02	16.63 ± 0.18	**	18.87 ± 0.93	***	10.60

Gen = genotype; WR = water regime; Gen * WR = interaction genotype × water regime. WUEL = water-use efficient inbred lines; WUIL = water-use inefficient inbred lines; HY = hybrids; HET = relative heterosis (%); C^1^ = mean contrast between water use efficient x inefficient inbred lines; C^2^ = mean contrast of hybrids versus inbred lines; * = significant difference at 5%; ** = significant difference at 1%; *** = significant difference at 0.1% by the *t* test. PH = plant height (cm); LA = leaf area (m^2^); SDW = stem dry weight (g); LDW = leaf dry weight (g); ShDW/RDW = shoot dry weight/root dry weight ratio; LW = leaf width (cm); LL = length of the leaf midrib (cm); and SD = stem diameter (mm).

**Table 2:** Summary of analysis of variances, mean estimates, standard deviations and relative heteroses of the decomposition of effects between genotypic groups (WUEL, WUIL, and HY) in popcorn for physiological characteristics evaluated under different water regimes.

Trait	Combined Analysis			Water-Stressed						Well-Watered				
	Gen	WR	Gen * WR	WUEL	WUIL	C^1^	HY	C^2^	HET	WUIL	C^1^	HY	C^2^	HET
A	**	***	ns	11.53 ± 2.31	7.31 ± 4.78	***	4.45 ± 1.50	***	−47.43	30.21 ± 5.45	ns	31.90 ± 6.30	ns	−0.78
*g_s_*	**	***	**	0.04 ± 0.02	0.03 ± 0.02	*	0.02 ± 0.02	*	−30.84	0.22 ± 0.08	ns	0.18 ± 0.02	**	−26.60
E	***	***	ns	0.83 ± 0.38	0.94 ± 0.81	ns	0.45 ± 0.15	**	−49.01	3.31 ± 0.82	ns	3.58 ± 0.70	ns	−1.50
VPD_leaf/air_	***	***	ns	2.01 ± 0.72	2.11 ± 0.13	*	2.20 ± 0.13	**	7.10	1.71 ± 0.19	ns	1.66 ± 0.20	ns	1.76
T_L_	*	**	ns	30.47 ± 0.77	30.89 ± 0.24	*	31.06 ± 0.50	ns	1.25	30.25 ± 0.61	ns	29.90 ± 0.62	ns	−0.61
F_v_/F_m_	ns	***	**	0.76 ± 0.02	0.78 ± 0.02	*	0.77 ± 0.01	ns	−0.95	0.79 ± 0.01	ns	0.80 ± 0.006	ns	0.98
SPAD index	*	**	ns	44.54 ± 1.23	45.48 ± 3.35	**	44.14 ± 1.28	ns	−1.86	50.93 ± 3.14	ns	49.59 ± 3.11	ns	−2.84
AWUE	***	**	*	3.35 ± 0.02	3.08 ± 0.04	*	3.60 ± 1.28	**	12.16	2.66 ± 0.55	*	3.80 ± 0.65	***	32.7
WF	***	*	*	0.3 ± 0.02	0.33 ± 0.06	ns	0.28 ± 0.02	**	−11.51	0.39 ± 0.08	*	0.27 ± 0.05	***	−24.7

Gen = genotype; WR = water regime; Gen * WR = genotype × water regime interaction. WUEL = water-use efficient inbred lines; WUIL = water use inefficient inbred lines; HY = hybrids; HET = relative heterosis (%); C^1^ = mean contrast of efficient versus inefficient inbred lines; C^2^ = mean contrast of hybrids versus inbred lines; * = significant difference at 5%; ** = significant difference at 1%; *** = significant difference at 0.1% by the *t* test. A = net CO_2_ assimilation rate (μmol.CO_2_.m^−2^.s^−1^); *gs* = stomatal conductance (mol.CO_2_.m^−2^.s^−1^); E = transpiration rate (mmol.H_2_0.m^−2^.s^−1^); VPD_leaf-air_ = leaf-to-air vapor pressure difference (kPa); T_L_ = leaf temperature (°C); F_v_/F_m_ = maximum quantum efficiency of photosystem II; SPAD index = chlorophyll concentration; AWUE = agronomic water use efficiency (g.L^−1^); and WF = water footprint (L.g^−1^).

**Table 3:** Summary of analysis of variances, mean estimates, standard deviations and relative heteroses of the decomposition of effects between genotypic groups (WUEL, WUIL, and HY) in popcorn for root traits evaluated under different water regimes.

Trait	Combined Analysis			Water-Stressed						Well-Watered					
	Gen	WR	Gen * WR	WUEL	WUIL	C^1^	HY	C^2^	HET	WUEL	WUIL	C^1^	HY	C^2^	HET
RL	*	**	ns	131.20 ± 18.44	124.2 ± 22.75	*	126.10 ± 20.01	ns	−1.26	149.80 ± 19.34	132.20 ± 24.16	*	155.30 ± 12.77	ns	10.17
RDW	***	***	**	6.86 ± 2.17	5.88 ± 1.19	**	6.90 ± 1.06	ns	4.03	13.21 ± 2.15	8.84 ± 3.59	ns	15.53 ± 4.42	***	20.12
SRA	**	ns	ns	42.30 ± 9.99	34,12 ± 9.81	ns	32.80 ± 10.12	ns	−13.39	31.50 ± 8.75	38.90 ± 8.50	*	33.10 ± 11.35	ns	−5.85
NSR	ns	**	ns	6.40 ± 1.51	6.22 ± 1.96	ns	5.60 ± 1.58	ns	−11.06	7.50 ± 1.73	7.50 ± 2.04	ns	8.20 ± 1.22	ns	9.53
SRD	ns	***	ns	4.23 ± 1.03	4.22 ± 1.45	ns	4.80 ± 1.99	ns	14.03	5.87 ± 1.11	5.73 ± 1.33	ns	6.33 ± 0.93	ns	9.19
CRA	***	ns	**	34.20 ± 3.32	35.75 ± 6.11	ns	26.70 ± 6.66	**	−23.21	31.60 ± 4.22	35.10 ± 5.94	*	27.60 ± 4.92	**	−2.56
NCR	ns	*	ns	17.40 ± 2.91	17.85 ± 3.89	ns	18.20 ± 4.61	ns	3.41	22.00 ± 3.13	19.20 ± 4.35	ns	19.90 ± 2.58	ns	−3.23
CRD	ns	ns	ns	5.07 ± 1.00	4.55 ± 1.19	ns	4.93 ± 0.44	ns	3.50	5.27 ± 0.83	4.67 ± 0.81	*	5.03 ± 0.76	ns	2.10

Gen = genotype; WR = water regime; Gen * WR = genotype × water regime interaction. WUEL = water-use efficient inbred lines; WUIL = water use inefficient inbred lines; HY = hybrids; HET = relative heterosis (%); C^1^ = mean contrast of efficient vs. inefficient inbred lines; C^2^ = mean contrast of hybrids vs. inbred lines; * = significant difference at 5%; ** = significant difference at 1%; *** = significant difference at 0.1% by the *t* test. RL = root length (cm); RDW = root dry weight (g); SRA = support root angle; NSR = number of support roots; SRD = support root density; CRA = crown root angle; NCR = number of crown roots; and CRD = crown root density.

## Data Availability

The raw data supporting the conclusions of this article will be made available by the authors, without undue reservation.
